# Ultrasound-Guided Botulinum Toxin A as an Adjunct to Intraoperative Fascial Traction in Incisional Hernia Repair: Registry-Based Cohort Study

**DOI:** 10.3390/diagnostics16050775

**Published:** 2026-03-04

**Authors:** Zaid Malaibari, Razaz Aldemyati, Henning Niebuhr, Halil Dag, Ferdinand Köckerling

**Affiliations:** 1 Department of Surgery, Faculty of Medicine, University of Tabuk, Tabuk 71491, Saudi Arabia; zmalaibari@ut.edu.sa; 2 Department of Surgery, Faculty of Medicine, King Abdulaziz University, Rabigh 21589, Saudi Arabia; 3 Hamburg Hernia Center, 20249 Hamburg, Germany; 4 Vivantes Humboldt Hospital Berlin, Academic Teaching Hospital of Charite`University Medicine, 13509 Berlin, Germany

**Keywords:** botulinum toxin A, ultrasound-guided injection, incisional hernia, abdominal wall reconstruction, primary fascial closure, intraoperative fascial traction, Herniamed registry, Fasciotens

## Abstract

**Background**: AchievTing primary fascial closure in complex incisional hernia repair can be challenging when abdominal wall compliance is reduced. Preoperative ultrasound-guided botulinum toxin A (BTA) is used as a chemical component relaxation adjunct, and intraoperative fascial traction (IFT) is a traction-based technique to facilitate medialization. This study assessed the association of adding BTA to a traction-treated cohort. **Methods**: Retrospective observational analysis of prospectively collected Herniamed Registry data from the Hamburg Hernia Center (1 February 2022–13 October 2025) was conducted. Elective incisional hernia repairs with IFT were included and stratified into BTA + IFT versus IFT-only. The primary outcome was primary fascial closure as documented in the registry. Categorical variables were compared using Fisher’s exact test. **Results**: A total of 81 patients were analyzed (BTA + IFT, *n* = 64; IFT-only, *n* = 17). Primary fascial closure was achieved in 51/64 (79.7%) in the BTA + IFT group and 8/17 (47.1%) in the IFT-only group (OR 4.3, 95% CI 1.22–15.84; *p* = 0.013). Mean operative time was similar (193 vs. 195 min). Mean length of stay was longer in the BTA + IFT group (8 vs. 5 days). Perioperative complications were recorded 8/64 (12.5%) in the BTA + IFT group and 0/17 (0.0%) in the IFT-only group. **Conclusions**: In traction-assisted incisional hernia repair, adjunctive preoperative ultrasound-guided BTA was associated with higher primary fascial closure rates compared with traction alone. Findings are hypothesis-generating due to non-randomized allocation and baseline differences between cohorts.

## 1. Introduction

Achieving primary fascial closure remains a central technical objective in complex incisional hernia repair. Large defects, loss-of-domain configurations, and reduced abdominal wall compliance can make fascial approximation challenging and may necessitate escalation strategies and adjuncts designed to improve tissue medialization and reduce closure tension.

Botulinum toxin A (BTA) induces temporary chemodenervation by inhibiting acetylcholine release at the neuromuscular junction, resulting in reversible muscle paralysis. Its application to abdominal wall reconstruction, often termed chemical component relaxation, was introduced to increase abdominal wall compliance and support fascial approximation. Early clinical experience in ventral and incisional hernia repair and subsequent studies reporting morphologic changes in the lateral abdominal wall musculature provided a mechanistic and clinical rationale for its use in selected complex cases [1,2,3].

Over the last decade, the clinical literature evaluating BTA as an adjunct to complex ventral/incisional hernia repair has expanded, and systematic reviews and meta-analyses have summarized technical aspects and outcomes. These syntheses suggest potential benefit for facilitating closure but emphasize substantial heterogeneity in patient selection, dosing, injection technique, timing, and concomitant operative strategies, limiting cross-study comparability and causal interpretation [4,5,6].

Modern BTA protocols are often performed under ultrasound guidance to support anatomical targeting of the external oblique, internal oblique, and transversus abdominis muscles for intramuscular injection [7,8]. In parallel, intraoperative techniques intended to facilitate gradual fascial medialization have emerged; intraoperative fascial traction (IFT) is a relatively recent strategy used to support tension-reduced closure [9]. A contemporary institutional protocol combining preoperative BTA and IFT has been described [10], but evidence specifically addressing the incremental association of adding BTA within a traction-treated cohort remains limited.

Therefore, the present study aimed to evaluate, within a contemporary single-center registry cohort, whether preoperative ultrasound-guided BTA in addition to IFT is associated with higher rates of primary fascial closure compared with IFT alone in elective incisional hernia repair, using prospectively collected data from the German Herniamed Registry at the Hamburg Hernia Center.

## 2. Materials and Methods

### 2.1. Study Design and Data Source

This study is a retrospective observational analysis of prospectively collected registry data. All included cases were recorded in the German Herniamed Registry. Written informed consent for the use of anonymized data for research purposes was obtained from all patients prior to surgery, in accordance with the registry participation process. The study was conducted in accordance with the Declaration of Helsinki; the Herniamed Registry has obtained ethical approval by the Ethics Committee Kanton St. Gallen (BASEC Nr. 2016-00123; date of approval 12 April 2016), and the Ethics Committee University of Tübingen (287/2017 BO2; date of approval 28 June 2017).

### 2.2. Study Period and Setting

Cases were identified at the Hamburg Hernia Center between 1 February 2022 and 13 October 2025 using standardized Herniamed registry filters and exports of anonymized patient data (incisional hernia module). Because structured registry fields/filters for IFT and preoperative BTA became available in February 2022, cases prior to 1 February 2022 could not be reliably identified using the predefined anonymized export filters and were therefore not included. The analysis included elective incisional hernia repairs in which intraoperative fascial traction (IFT; Fasciotens^®^ system, Fasciotens GmbH, Essen, Germany) was applied, with or without preoperative ultrasound-guided BTA injections.

### 2.3. Eligibility Criteria

Inclusion Criteria

Patients were eligible for inclusion if they met all of the following criteria:

1. Adult patients (≥18 years) undergoing elective incisional hernia repair.
2. Incisional hernia location classified as midline or lateral.
3. Use of intraoperative fascial traction (Fasciotens^®^ system) during hernia repair.
4. Availability of the relevant operative and perioperative data fields in the registry export.

Exclusion Criteria

Patients were excluded if any of the following criteria applied:

1. Emergency hernia repair.
2. Primary ventral hernias (i.e., non-incisional abdominal wall hernias).
3. Parastomal hernias.
4. Use of progressive pneumoperitoneum.
5. Hernias not fulfilling the predefined operative approach criteria of the study.

### 2.4. Exposure Groups

Patients were stratified into two groups based on receipt of preoperative BTA:

• BTA + IFT group: preoperative ultrasound-guided BTA followed by intraoperative fascial traction.
• IFT-only group: intraoperative fascial traction without BTA.

### 2.5. Preoperative Ultrasound-Guided Botulinum Toxin A Protocol

Preoperative chemical component relaxation using BTA was performed according to the standardized institutional protocol of the Hamburg Hernia Center, which has been previously described [10]. BTA was routinely administered four weeks prior to surgery as an outpatient, ultrasound-guided procedure. All BTA cases followed the same standardized institutional protocol (uniform total dose, dilution, target muscles, and injection sites) and were performed under real-time ultrasound guidance by surgeons trained in this protocol. Although procedural variability cannot be fully excluded, standardization was intended to minimize it.

**Figure 1:**
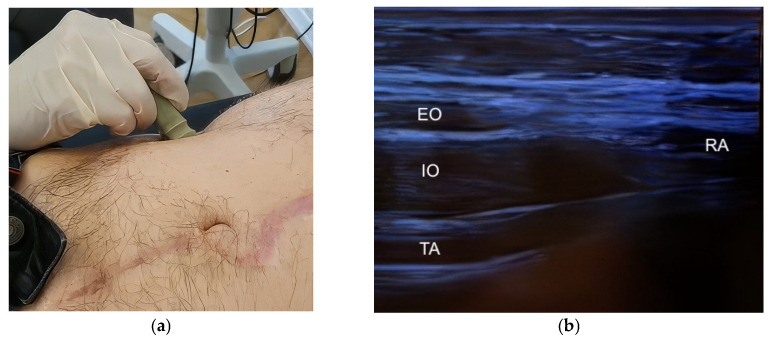
Transverse ultrasound orientation for anatomical targeting prior to BTA injection. (**a**) Representative photograph showing placement of a linear small-parts transducer (frequency 9–12 MHz) in transverse orientation on the anterolateral abdominal wall to establish anatomical orientation before injection. (**b**) Representative transverse ultrasound image demonstrating the layered lateral abdominal wall musculature and its relation to the rectus abdominis: external oblique (EO), internal oblique (IO), transversus abdominis (TA), and rectus abdominis (RA). Abbreviations: EO, external oblique; IO, internal oblique; TA, transversus abdominis; RA, rectus abdominis.

**Figure 2:**
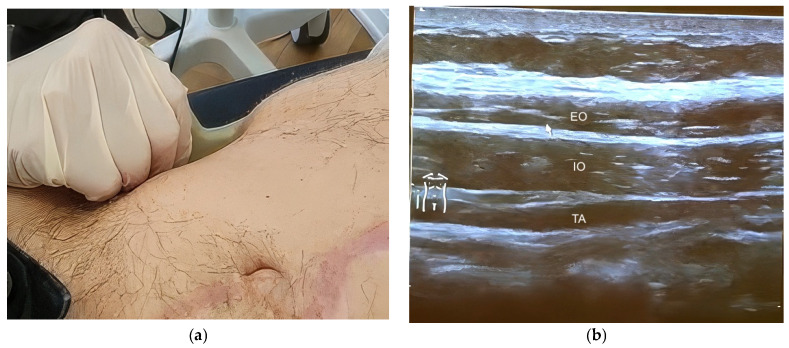
Longitudinal ultrasound orientation for layer delineation prior to injection. (**a**) Representative photograph showing placement of a linear ultrasound transducer in longitudinal orientation on the anterolateral abdominal wall after transverse orientation has been established. (**b**) Representative longitudinal ultrasound image demonstrating the layered lateral abdominal wall musculature: external oblique (EO), internal oblique (IO), and transversus abdominis (TA). Abbreviations: EO, external oblique; IO, internal oblique; TA, transversus abdominis.

Ultrasound examination of the lateral abdominal wall was performed to identify the muscular layers, including the external oblique (EO), internal oblique (IO), and transversus abdominis (TA) muscles. Muscle identification and spatial orientation relative to the rectus abdominis muscle were achieved using a linear small-parts transducer (frequency 9–12 MHz) in transverse orientation (Figure 1a,b). A longitudinal probe orientation was subsequently used to delineate the individual muscle layers prior to injection (Figure 2a,b).

**Figure 3:**
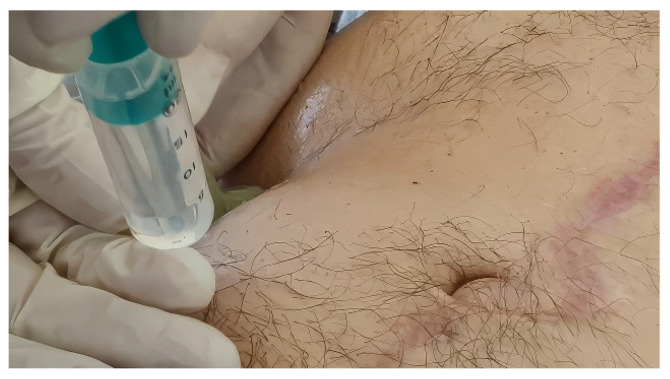
Ultrasound-guided injection setup (representative image). Representative photograph illustrating the injection step of the outpatient ultrasound-guided BTA procedure, showing needle–syringe positioning adjacent to the linear transducer during intramuscular injection into the lateral abdominal wall under real-time ultrasound guidance.

Botulinum toxin A (Botox^®^, Allergan, Dublin, Ireland) was administered at a total dose of 200 IU, diluted in 40 mL of 0.9% saline, with 100 IU in 20 mL injected per side (Figure 3). Under real-time ultrasound guidance, injections were delivered into the EO, IO, and TA muscles at three predefined injection sites per side, with the three muscle layers injected sequentially at each site. Correct intramuscular deposition was confirmed sonographically by visible expansion and spread within the targeted muscle layer (Supplementary Video S1).

Following the procedure, patients were observed briefly in the outpatient setting and subsequently discharged.

### 2.6. Surgical Approach and Intraoperative Fascial Traction

Hernia repair was performed predominantly using retromuscular sublay techniques (retrorectus repair in line with Rives–Stoppa principles) with mesh augmentation in sublay position, in accordance with institutional practice. Where appropriate, retromuscular sublay reconstruction was performed using Mini- or Less-Open Sublay techniques (MiLOS), which adhere to the same anatomical and reconstructive principles. In a minority of cases, alternative operative approaches were used as recorded in the registry export, reflecting individualized intraoperative decision-making.

The primary surgical objective was tension-reduced primary fascial closure. When low-tension fascial approximation was not achievable after standard preparation, IFT was applied using the Fasciotens^®^ hernia traction system. IFT was applied according to surgeon’s judgment in cases where low-tension primary fascial approximation remained unattainable after standard preparation, including selected lateral defects. Intraoperatively, traction sutures were placed lateral to the anterior rectus sheath margins (or the corresponding fascial margins depending on defect location), and controlled traction was applied for approximately 30 min, with intermittent retightening to facilitate gradual medialization of the abdominal wall tissues. If fascial approximation remained insufficient after fascial traction, additional escalation strategies were considered. Component separation techniques (anterior or posterior; e.g., transversus abdominis release) were considered when additional medialization was required to achieve low-tension fascial continuity, whereas bridging mesh repair was reserved for situations in which fascial continuity could not be achieved safely or was deemed technically infeasible (based on intraoperative judgment) despite traction and/or release manoeuvers [10].

### 2.7. Outcome Measures

Primary Outcome

The primary outcome was primary fascial closure. For this analysis, primary fascial closure was defined as restoration of fascial continuity by approximation and suturing of the fascial edges. Cases recorded as “no defect closure” were classified as no closure, including situations in which fascial continuity was not achieved (e.g., bridging repair/no approximation). Closure achieved after intraoperative escalation (e.g., component separation techniques including TAR) was still classified as primary fascial closure because the endpoint reflects final intraoperative fascial continuity rather than the specific technique used to achieve it.

Secondary Outcomes

• Operative time (minutes).
• Length of hospital stay (days).
• Postoperative complications occurring within the registry-defined perioperative window, including surgical site occurrences, surgical site infections and reoperation rate, where available.

### 2.8. Statistical Analysis

Statistical analyses were performed using R (version 4.5.2; R Core Team, R Foundation for Statistical Computing, Vienna, Austria, 2025) [11]. Categorical variables were summarized as absolute numbers and percentages and compared using Fisher’s exact test, given the sample size distribution. Continuous variables were summarized using means and compared descriptively between groups. Analyses were performed on available data; denominators vary by variable; no imputation was performed.

Results are reported primarily as absolute differences and effect estimates. *p*-values are provided for descriptive purposes where appropriate, without reliance on predefined significance thresholds. No multivariable analyses were performed.

### 2.9. Declaration of Generative AI Use

The authors declare that Generative AI was used in the preparation of this manuscript. ChatGPT (GPT-5.2, OpenAI, San Francisco, CA, USA) was used to refine text and improve clarity and language. It was not used for data extraction, data generation, statistical analysis, or scientific interpretation. All references, results, and analyses were provided and verified by the authors, who reviewed all AI-assisted text and take full responsibility for the content.

## 3. Results

### 3.1. Study Population

During the study period from 1 February 2022 to 13 October 2025, a total of 81 patients met the predefined inclusion criteria and were included in the analysis. Of these, 64 patients underwent preoperative ultrasound-guided BTA followed by intraoperative fascial traction (BTA + IFT group), and 17 patients underwent intraoperative fascial traction without BTA (IFT-only group).

All included patients underwent elective incisional hernia repair (midline or lateral) with application of intraoperative fascial traction as part of the reconstructive strategy.

### 3.2. Patients and Hernia Characteristics

Baseline demographic, clinical, and defect characteristics are summarized in Table 1, Table 2 and Table 3. The BTA + IFT group comprised patients with larger defect dimensions and defect area categories, whereas the IFT-only group demonstrated a broader distribution across smaller defect size categories. Detailed topographic European Hernia Society (EHS) location sub-classification (midline M1–M5 and lateral L1–L4, as documented in the registry export) is provided in Supplementary Table S1. Operative approaches recorded in the registry are summarized in Supplementary Table S2; retromuscular sublay-plane techniques (Rives–Stoppa level) predominated in both cohorts.

### 3.3. Primary Outcome: Primary Fascial Closure

Primary fascial closure was achieved in 51/64 patients (79.7%) in the BTA + IFT group and 8/17 patients (47.1%) in the IFT-only group. Primary fascial closure was defined as specified in Section 2.7. This corresponded to an odds ratio of 4.3 (95% CI 1.22–15.84; Fisher’s exact test *p* = 0.013). Closure outcomes by group are summarized in Table 4.

### 3.4. Secondary Outcomes

Secondary outcomes are summarized in Table 5. Operative time was similar between groups. The length of hospital stay was longer in the BTA + IFT group. Postoperative complications within the registry-defined perioperative window were recorded only in the BTA + IFT group; complication categories (not mutually exclusive) are detailed in Table 5.

## 4. Discussion

### 4.1. Principal Findings

In this single-center registry analysis of elective incisional hernia repairs performed between 2022 and 2025 with intraoperative fascial traction, the addition of preoperative ultrasound-guided BTA was associated with a higher rate of primary fascial closure compared with intraoperative fascial traction alone (Table 4).

Given the surgeon-directed allocation and the substantial baseline differences in defect morphology and complexity between cohorts, these findings should be interpreted as descriptive associations rather than causal effects. The BTA + IFT cohort represents a more complex population in routine practice, which likely contributes to the observed differences in perioperative outcomes and resource use.

Secondary outcomes showed similar operative times between groups, while length of stay and perioperative complications were higher in the BTA + IFT cohort (Table 5). These secondary outcome differences should be interpreted cautiously and likely reflect overall case complexity and care pathways rather than an isolated effect of BTA.

### 4.2. Interpretation Within a Traction-Based Closure Strategy

The study evaluates BTA within a defined institutional treatment approach: ultrasound-guided chemical component relaxation performed preoperatively [12,13,14], and intraoperative fascial traction applied when low-tension approximation was not achievable after standard preparation [9,10]. Within this framework, BTA represents a preoperative adjunct intended to increase abdominal wall compliance and support tension-reduced fascial approximation [15,16].

Prior observational and propensity-based comparative studies of preoperative BTA in complex abdominal wall reconstruction have reported an association with improved closure-related endpoints—most commonly higher rates of primary fascial closure and/or reduced need for escalation strategies—while emphasizing that effect estimates are sensitive to case selection and concomitant reconstructive techniques [13,14,17]. By restricting the analysis to cases managed with IFT, the present study focuses on the association of adding BTA within a traction-assisted closure strategy, rather than comparing fundamentally different reconstructive pathways.

Because both cohorts received intraoperative traction, the present analysis estimates the association of adding BTA within this approach; however, mechanistic attribution and causal inference are limited by surgeon-directed allocation and baseline imbalance (Table 1 and Table 2). Progressive pneumoperitoneum (PPP) was not part of the institutional protocol and was excluded; therefore, these findings apply to a strategy combining ultrasound-guided BTA and traction without PPP [18,19,20].

### 4.3. Ultrasound-Guided Preoperative Intervention

In this cohort, BTA was administered as a standardized outpatient procedure under ultrasound guidance to identify the lateral abdominal wall muscle layers and guide intramuscular injection. Ultrasound provides real-time soft-tissue visualization for needle guidance without ionizing radiation and is widely accessible in outpatient and perioperative settings [8]. CT-guided injection represents an alternative image-guided approach in selected settings [21]. Although ultrasound is operator-dependent, a predefined injection protocol and continuous visualization of target anatomy support procedural standardization by facilitating consistent anatomical targeting and intramuscular deposition.

### 4.4. Strengths and Limitations

A key strength of this study is the analysis of prospectively documented cases from a specialized abdominal wall reconstruction center, with a standardized ultrasound-guided BTA protocol and a consistent traction-based operative strategy across the included cohort. The primary endpoint—primary fascial closure—is clinically relevant and directly reflects the reconstructive objective in complex incisional hernia repair.

Because registry capture of IFT/BTA variables was implemented from February 2022, earlier cases are not represented in the export.

Interpretation is limited by the retrospective, non-randomized design with surgeon-directed allocation of BTA and substantial baseline imbalance in defect morphology between cohorts, resulting in a high risk of confounding by indication and precluding causal inference. The IFT-only cohort was small, reducing precision and yielding wide confidence intervals. For rare events such as postoperative complications, zero events in the IFT-only cohort further limits precision and interpretability of between-group comparisons. The present analysis addresses intraoperative feasibility (primary fascial closure) and perioperative outcomes; assessment of long-term recurrence and patient-reported outcomes warrants dedicated studies with standardized follow-up. In addition, the distribution of defect topography differed between groups, with more frequent lateral location coding in the IFT-only cohort, which may influence closure feasibility and complicate direct comparison. Finally, intraoperative fascial traction is a comparatively recent technique and its indications and application may still be evolving; therefore, unmeasured differences in selection thresholds and operative decision-making may have contributed to the observed group differences. No multivariable adjustment was performed, and the findings should therefore be interpreted as descriptive and hypothesis-generating. Longer hospital stay and more complex perioperative courses in the BTA + IFT cohort may have increased the opportunity for detecting and documenting complications.

### 4.5. Future Directions

Future work should prioritize explicit, morphology-based selection criteria for preoperative ultrasound-guided BTA in traction-assisted reconstruction (e.g., defect dimensions, topographic classification, and abdominal cavity/hernia sac volumetry) and evaluate outcomes using prespecified perioperative endpoints in designs that better address confounding. In parallel, an ongoing randomized, double-blind, placebo-controlled trial is currently recruiting to evaluate image-guided BOTOX^®^ versus placebo for large ventral hernia repair with primary fascial closure as the primary endpoint (ClinicalTrials.gov, NCT07220382) [22]. In addition, future studies should incorporate standardized follow-up to assess longer-term recurrence and patient-reported outcomes to contextualize perioperative feasibility. Future controlled and/or multicenter studies should prespecify analytic strategies such as multivariable adjustment or propensity-based methods to better address confounding by indication.

## 5. Conclusions

In this single-center registry analysis of elective incisional hernia repairs performed with intraoperative fascial traction, the addition of preoperative ultrasound-guided botulinum toxin A was associated with a higher rate of primary fascial closure compared with intraoperative fascial traction alone. Given surgeon-directed allocation and substantial baseline differences in defect morphology and complexity between cohorts, these findings should be interpreted as descriptive and hypothesis-generating rather than causal. Future controlled and/or multicenter studies with standardized follow-up and prespecified analytic strategies are needed to define the benefit–risk balance of adding botulinum toxin A to traction-assisted reconstruction.

## Figures and Tables

**Table 1:** Demographics and perioperative risk profile.

Characteristic	BTA + IFT (*n* = 64)	IFT-Only (*n* = 17)
**Sex**		
Male	32 (50.0%)	13 (76.5%)
Female	32 (50.0%)	4 (23.5%)
**Age (years)**		
<60	32 (50.0%)	5 (29.4%)
60–69	19 (29.7%)	7 (41.2%)
≥70	13 (20.3%)	5 (29.4%)
**BMI category**		
Normal weight	4 (6.3%)	5 (29.4%)
Overweight	21 (32.8%)	7 (41.2%)
Obesity	29 (45.3%)	3 (17.6%)
Morbid obesity	10 (15.6%)	2 (11.8%)
**ASA class**		
ASA I	1 (1.6%)	2 (11.8%)
ASA II	23 (35.9%)	6 (35.3%)
ASA III	40 (62.5%)	9 (52.9%)
ASA IV	0 (0.0%)	0 (0.0%)

Notes: Values are *n* (%) unless otherwise stated. Age categories were collapsed for descriptive presentation to avoid sparse cells in the IFT-only group. Abbreviations: ASA, American Society of Anesthesiologists; BMI, body mass index; BTA, botulinum toxin A; IFT, intraoperative fascial traction.

**Table 2:** Hernia history and defect morphology.

Characteristic	BTA + IFT (*n* = 64)	IFT-Only (*n* = 17)
**Incisional hernia repair status**		
Primary repair	39 (60.9%)	13 (76.5%)
Recurrent repair	25 (39.1%)	4 (23.5%)
**Defect maximal length (cm)**		
<4	0 (0.0%)	2 (11.8%)
4–10	2 (3.1%)	5 (29.4%)
>10–15	9 (14.1%)	5 (29.4%)
>15	53 (82.8%)	5 (29.4%)
**Defect maximal width (cm)**		
<4	0 (0.0%)	5 (29.4%)
4–10	0 (0.0%)	3 (17.6%)
>10–15	24 (37.5%)	8 (47.1%)
>15	41 (64.1%)	1 (5.9%)
**Defect area (cm^2^)**		
<100	0 (0.0%)	7 (41.2%)
100–200	7 (10.9%)	5 (29.4%)
>200	57 (89.1%)	5 (29.4%)

Notes: Values are *n* (%). Abbreviations: BTA, botulinum toxin A; IFT, intraoperative fascial traction.

**Table 3:** Recorded risk factors.

Risk Factor	BTA + IFT (*n* = 56 *)	IFT-Only (*n* = 11 *)
COPD/asthma	43 (76.8%)	3 (27.3%)
Diabetes mellitus	35 (62.5%)	4 (36.4%)
Smoking	11 (19.6%)	5 (45.5%)
Immunosuppression	3 (5.4%)	2 (18.2%)
Aortic aneurysm	2 (3.6%)	1 (9.1%)
Corticosteroid use	1 (1.8%)	0 (0.0%)
Coagulation disorder	10 (17.9%)	0 (0.0%)
Antiplatelet therapy stopped < 7 days	12 (21.4%)	2 (18.2%)
NOAC stopped < 2 days	5 (8.9%)	1 (9.1%)
Liver cirrhosis	0 (0.0%)	1 (9.1%)

* Denominator reflects patients with documented risk-factor data in the registry export (BTA + IFT: *n* = 57; IFT-only: *n* = 11). Percentages are calculated using these denominators. Risk factors are not mutually exclusive; individual patients may have more than one recorded risk factor. Therefore, the sum of risk-factor counts exceeds the number of patients. Abbreviations: COPD, chronic obstructive pulmonary disease; NOAC, non-vitamin K antagonist oral anticoagulant.

**Table 4:** Primary outcome (primary fascial closure).

Outcome	BTA + IFT (*n* = 64)	IFT-Only (*n* = 17)
Primary fascial closure (yes)	51 (79.7%)	8 (47.1%)
Primary fascial closure (no)	13 (20.3%)	9 (52.9%)

Notes: Values are *n* (%). Abbreviations: BTA, botulinum toxin A; IFT, intraoperative fascial traction.

**Table 5:** Secondary outcomes.

Secondary Outcome	BTA + IFT (*N* = 64)	IFT-Only (*N* = 17)
Operative time, mean (min)	193	195
Length of hospital stay, mean (days)	8	5
Reoperation, *n* (%)	5 (7.8%)	0
Any postoperative complication, *n* (%)	8 (12.5%)	0 (0.0%)
Postoperative bleeding, count	1	0
Wound-healing disorder, count	3	0
Seroma, count	3	0
Infection, count	4	0

Notes: Values are *n* (%) unless otherwise stated. “Any postoperative complication” reflects the number of cases with ≥1 postoperative complication recorded in the registry. Specific complication categories are not mutually exclusive; therefore, category totals may exceed the number of cases with any complication and may also exceed *N*. Abbreviations: BTA, botulinum toxin A; IFT, intraoperative fascial traction.

## Data Availability

The data presented in this study are available on request from the corresponding author due to the required permissions and approval by the data custodian/registry.

## Supplementary Materials

The following supporting information can be downloaded at https://www.mdpi.com/article/10.3390/diagnostics16050775/s1, Table S1: EHS topographic location sub-classification (as documented in the registry); Table S2: Operative approach recorded in the registry by cohort; Video S1: Sonographic confirmation of intramuscular injectate deposition during ultrasound-guided BTA injection.

## Abbreviations

The following abbreviations are used in this manuscript:

BTA	Botulinum toxin A
IFT	intraoperative fascial traction
EO	external oblique
IO	internal oblique
TA	transversus abdominis
MiLOS	Mini- or Less-Open Sublay techniques
COPD	chronic obstructive pulmonary disease
NOAC	non-vitamin K antagonist oral anticoagulant.
EHS	European Hernia Society

## References

[1] Ibarra-Hurtado T.R., Nuño-Guzmán C.M., Echeagaray-Herrera J.E., Robles-Vélez E., González-Jaime J.D.J. (2009). Use of botulinum toxin type A before abdominal wall hernia reconstruction. World J. Surg..

[2] Ibarra-Hurtado T.R., Nuño-Guzmán C.M., Miranda-Díaz A.G., Troyo-Sanromán R., Navarro-Ibarra R., Bravo-Cuéllar L. (2014). Effect of botulinum toxin type A in lateral abdominal wall muscles thickness and length of patients with midline incisional hernia secondary to open abdomen management. Hernia.

[3] Zendejas B., Khasawneh M.A., Srvantstyan B., Jenkins D.H., Schiller H.J., Zielinski M.D. (2013). Outcomes of chemical component paralysis using botulinum toxin for incisional hernia repairs. World J. Surg..

[4] Timmer A.S., Claessen J.J.M., Atema J.J., Rutten M.V.H., Hompes R., Boermeester M.A. (2021). A systematic review and meta-analysis of technical aspects and clinical outcomes of botulinum toxin prior to abdominal wall reconstruction. Hernia.

[5] Dias E.R.M., Rondini G.Z., Amaral P.H.F., Macret J.Z., Carvalho J.P.V., Pivetta L.G.A., Malheiros C.A., Roll S. (2023). Systematic review and meta-analysis of the pre-operative application of botulinum toxin for ventral hernia repair. Hernia.

[6] Barretto V.R.D., de Oliveira J.G.R., Brim A.C.S., Araújo R.B.S., Barros R.A., Romeo A.L.B. (2024). Botulinum toxin A in complex incisional hernia repair: A systematic review. Hernia.

[7] Hipolito Canario D.A., Isaacson A.J., Martissa J.A., Stewart J.K. (2021). Ultrasound-guided chemical component separation with botulinum toxin A prior to surgical hernia repair. J. Vasc. Interv. Radiol..

[8] Kurumety S., Walker A., Samet J., Grant T., Dumanian G.A., Deshmukh S. (2021). Ultrasound-guided lateral abdominal wall botulinum toxin injection before ventral hernia repair: A review for radiologists. J. Ultrasound Med..

[9] Woeste G., Dascalescu S., Wegner F., Meier H., Sardoschau N., Kiehle A., Dag H., Malaibari Z., Niebuhr H. (2025). Follow-up of complex hernia repair with intraoperative fascial traction. Hernia.

[10] Niebuhr H., Wegner F., Dag H., Reinpold W., Woeste G., Köckerling F. (2024). Preoperative botolinum toxin A (BTA) and intraoperative fascial traction (IFT) in the management of complex abdominal wall hernias. Hernia.

[11] R Core Team (2025). R: A Language and Environment for Statistical Computing.

[12] Motz B.M., Schlosser K.A., Heniford B.T. (2018). Chemical components separation: Concepts, evidence, and outcomes. Plast. Reconstr. Surg..

[13] Deerenberg E.B., Shao J.M., Elhage S.A., Lopez R., Ayuso S.A., Augenstein V.A., Heniford B.T. (2021). Preoperative botulinum toxin A injection in complex abdominal wall reconstruction—A propensity-score matched study. Am. J. Surg..

[14] Marturano M.N., Ayuso S.A., Ku D., Raible R., Lopez R., Scarola G.T., Gersin K., Colavita P.D., Augenstein V.A., Heniford B.T. (2023). Preoperative botulinum toxin A (BTA) injection versus component separation techniques (CST) in complex abdominal wall reconstruction (AWR): A propensity-score matched study. Surgery.

[15] Claessen J.J.M., Timmer A.S., Hemke R., Atema J.J., Hompes R., Boermeester M.A., Rutten M.V.H. (2023). A computed tomography study investigating the effects of botulinum toxin injections prior to complex abdominal wall reconstruction. Hernia.

[16] Amaral P.H.F., Macret J.Z., Dias E.R.M., Carvalho J.P.V., Pivetta L.G.A., Ribeiro H.B., Franciss M.Y., Silva R.A., Malheiros C.A., Roll S. (2024). Volumetry after botulinum toxin A: The impact on abdominal wall compliance and endotracheal pressure. Hernia.

[17] de Jong D.L.C., Wegdam J.A., van der Wolk S., Nienhuijs S.W., de Vries Reilingh T.S. (2024). Prevention of component separation in complex abdominal wall surgery by Botox prehabilitation: A propensity-matched study. Hernia.

[18] Bueno-Lledó J., Torregrosa A., Jiménez R., Pastor P.G. (2018). Preoperative combination of progressive pneumoperitoneum and botulinum toxin type A in patients with loss of domain hernia. Surg. Endosc..

[19] van Rooijen M.M.J., Yurtkap Y., Allaeys M., Ibrahim N., Berrevoet F., Lange J.F. (2021). Fascial closure in giant ventral hernias after preoperative botulinum toxin A and progressive pneumoperitoneum: A systematic review and meta-analysis. Surgery.

[20] Giuffrida M., Biolchini F., Capelli P., Banchini F., Perrone G. (2024). Botulinum toxin and progressive pneumoperitoneum in loss of domain ventral hernias: A systematic review. J. Abdom. Wall Surg..

[21] Jahangiri Y., Goldsmith D., Banks-Venegoni A., Fritz G., Zambito G., Jiao A., King L., Castle J., Quasem K., Morrison J. (2025). Effectiveness of pre-operative chemical component separation with computed tomography-guided intramuscular injection of onabotulinumtoxinA in outcomes of large complex incisional ventral abdominal hernia repair: A propensity score-weighted comparative analysis. Hernia.

[22] ClinicalTrials.gov Preoperative BOTOX^®^ Injection for Large Ventral Hernia Repair (ClinicalTrials.gov Identifier: NCT07220382). NCT07220382.

