# Supplementary material for: Ultrasound-Guided Botulinum Toxin A as an Adjunct to Intraoperative Fascial Traction in Incisional Hernia Repair: Registry-Based Cohort Study
Source: Diagnostics (Basel). 2026 Mar 4;16(5):775. doi: 10.3390/diagnostics16050775 (PMC12985070; doi:10.3390/diagnostics16050775)
Supplement: Supplementary file 1 [file diagnostics-16-00775-s001.zip › diagnostics-4122960-supplementary.pdf]

Supplementary Table S1. EHS topographic location sub-classification (as documented in the registry)

EHS location category	BTA + IFT (n = 65)	IFT-only (n = 17)
**Midline (M)**		
M1 Subxiphoidal	34 (52.3%)	4 (23.5%)
M2 Epigastric	55 (84.6%)	15 (88.2%)
M3 Umbilical	59 (90.8%)	15 (88.2%)
M4 Infraumbilical	55 (84.6%)	7 (41.2%)
M5 Suprapubic	25 (38.5%)	1 (5.9%)
**Lateral (L)**		
L1 Subcostal	1 (1.5%)	2 (11.8%)
L2 Flank	5 (7.7%)	2 (11.8%)
L3 Iliac	0 (0.0%)	1 (5.9%)
L4 Lumbar	0 (0.0%)	0 (0.0%)

Notes: Values are n (% of patients). Categories are not mutually exclusive; multiple locations may be recorded per patient.

Abbreviations: EHS, European Hernia Society; BTA, botulinum toxin A; IFT, intraoperative fascial traction.

Supplementary Video S1. Sonographic confirmation of intramuscular injectate deposition during ultrasound-guided BTA injection.

Representative ultrasound clip demonstrating visible focal expansion/spread of injectate within the targeted lateral abdominal wall muscle layer during injection, used as real-time confirmation of intramuscular deposition.

Supplementary Table S2. Operative approach recorded in the registry by cohort

OPERATIVE APPROACH (REGISTRY)	BTA + IFT (N=64)	IFT-ONLY (N=17)
**RETROMUSCULAR SUBLAY PLANE (RIVES–STOPPA LEVEL), TOTAL**	**61**	**16**
– OPEN SUBLAY	*55*	*1*
– MILOS	*6*	*9*
– EMILOS	*0*	*6*
**COMPONENT SEPARATION**	**2**	**0**
**OTHER**	**1**	**1**

Retromuscular sublay plane total = open sublay + MILOS + EMILOS.
